# Inflammatory Bowel Disease: Autoimmune or Immune-mediated Pathogenesis?

**DOI:** 10.1080/17402520400004201

**Published:** 2004

**Authors:** Zhonghui Wen, Claudio Fiocchi

**Affiliations:** Division of Gastroenterology University Hospitals of Cleveland Case Western Reserve University School of Medicine Cleveland OH 44106-4952 USA

## Abstract

The pathogenesis of Crohn's disease (CD) and ulcerative colitis (UC), the two main forms of inflammatory bowel disease (IBD), is still unclear, but both autoimmune and immune-mediated phenomena are involved. Autoimmune phenomena include the presence of serum and mucosal autoantibodies against intestinal epithelial cells in either form of IBD, and against human tropomyosin fraction five selectively in UC. In addition, perinuclear antineutrophil cytoplasmic antibodies (pANCA) are common in UC, whereas antibodies against *Saccharomyces cerevisiae* (ASCA) are frequently found in CD. Immune-mediate phenomena include a variety of abnormalities of humoral and cell-mediated immunity, and a generalized enhanced reactivity against intestinal bacterial antigens in both CD and UC. It is currently believed that loss of tolerance against the indigenous enteric flora is the central event in IBD pathogenesis. Various complementary factors probably contribute to the loss of tolerance to commensal bacteria in IBD. They include defects in regulatory T-cell function, excessive stimulation of mucosal dendritic cells, infections or variants of proteins critically involved in bacterial antigen recognition, such as the products of CD-associated *NOD2/CARD15* mutations.


*Clinical & Developmental Immunology*, September/December 2004, Vol. 11 (3/4), pp. 195–204

Taylor & Francis healthsciences

### Inflammatory Bowel Disease: Autoimmune or Immune-mediated Pathogenesis?

ZHONGHUI WEN and CLAUDIO FIOCCHI*

*Division of Gastroenterology, University Hospitals of Cleveland, Case Western Reserve University School of Medicine, Cleveland, OH 44106-4952, USA*

The pathogenesis of Crohn's disease (CD) and ulcerative colitis (UC), the two main forms of inflammatory bowel disease (IBD), is still unclear, but both autoimmune and immune-mediated phenomena are involved. Autoimmune phenomena include the presence of serum and mucosal autoantibodies against intestinal epithelial cells in either form of IBD, and against human tropomyosin fraction five selectively in UC. In addition, perinuclear antineutrophil cytoplasmic antibodies (pANCA) are common in UC, whereas antibodies against *Saccharomyces cerevisiae* (ASCA) are frequently found in CD. Immune-mediate phenomena include a variety of abnormalities of humoral and cell-mediated immunity, and a generalized enhanced reactivity against intestinal bacterial antigens in both CD and UC. It is currently believed that loss of tolerance against the indigenous enteric flora is the central event in IBD pathogenesis. Various complementary factors probably contribute to the loss of tolerance to commensal bacteria in IBD. They include defects in regulatory T-cell function, excessive stimulation of mucosal dendritic cells, infections or variants of proteins critically involved in bacterial antigen recognition, such as the products of CD-associated *NOD2/CARD15* mutations.

*Keywords*: Inflammatory bowel disease; Crohn's disease; Ulcerative colitis; Intestinal flora; Tolerance

### INTRODUCTION

Chronic inflammatory diseases where an external infectious or noxious agent is not readily identified as the direct cause of the associated pathology are usually categorized as autoimmune or immune-mediated conditions. This implies a dominant role of the immune system in triggering and maintaining an inflammatory response against known or unknown antigens that are part of the host or intimately associated with it. In reality, chronic disabling inflammatory diseases of uncertain etiology are the result of highly complex and intimately integrated biological networks that also include genetic, environmental and neuroendocrine components (Straub and Besedovsky, 2003). This concept also applies to Crohn's disease (CD) and ulcerative colitis (UC), two chronic inflammatory conditions of the gastrointestinal tract that are collectively known as inflammatory bowel diseases (IBD). There is considerable evidence demonstrating that immunopathogenic events are distinct in CD and UC (Fiocchi, 1998; Podolsky, 2002; Bouma and Strober, 2003), but these entities also share plausible pathogenic factors such as dietary antigens and the enteric commensal flora. The latter, in particular, has received a great deal of attention in the last decade due to mounting evidence that it may function as a "self-antigen" in IBD, a notion that will be discussed later on. Still in regard to the role of the enteric flora, when discussing organ-specific autoimmune or immune-mediated pathogenesis, a fundamental concept that cannot be overemphasized is that both CD and UC occur in a non sterile, "dirty" tissue environment, unlike any other type of autoimmune or immune-mediated disorder that develops in sterile, "clean" tissues, such as the joint in rheumatoid arthritis, the nervous system in multiple sclerosis, the thyroid in autoimmune thyroiditis, the bile ducts in primary biliary cirrhosis, the microcirculation in systemic lupus erythematosus, and so on. This crucial difference between IBD and other immunological conditions is at the core of the present review, and will allow us to consider the intriguing idea of the normal enteric flora not as an external environmental factor but an intrinsic, "self-component" of the intestine-specific immune response occurring in CD and UC.

### AUTOIMMUNE EVENTS IN IBD

Classical criteria defining an autoimmune disease include the demonstration of B-cell clones producing polyreactive

*Corresponding author. Tel.: +1-216-368-1669. Fax: +1-216-368-1674. E-mail: cxf18@po.cwru.edu

ISSN 1740-2522 print/ISSN 1740-2530 online © 2004 Taylor & Francis Ltd
DOI: 10.1080/17402520400004201

antibodies, antibodies specific for autoantigens, and pathogenic autoantibodies, T-cell clones that are specific for autoantigens and can transfer autoimmune disease, the precise identification of organ-specific autoantigens, and the reproduction of autoimmune diseases in experimental animal models (Rose and Bona, 1993). In addition, we know that autoimmune diseases are conditioned by genetic and environmental factors (Marrack *et al.*, 2001), and among the latter a variety of infectious agents have been demonstrated to possess the ability to induce or activate autoreactive immune cells (Wucherpfennig, 2001). Molecular mimicry has been invoked as one of the mechanisms responsible for the activation of autoreactive cells by microbial peptides that have structural similarities to self-peptides (Wucherpfennig, 2001), but there is also evidence that antigenically unrelated infections or specific inflammatory signals can result in autoaggressiveness and induction of organ-specific autoimmunity, including in the gut (Vezys and Lefrançois, 2002). Neither CD nor UC fulfill all or most of the criteria for classical autoimmunity. On the other hand, there is evidence that autoimmune reactivity does occur in IBD.

### Autoantibodies

The first reasonable indication that the immune system was involved in the pathogenesis of IBD emerged in the late 1950s and early 1960s with the demonstration of autoantibodies and cytotoxic leukocytes for colonic epithelial cells in UC patients (Broberger and Perlmann, 1959; Perlmann and Broberger, 1963). Soon after, serum antibodies against colonic epithelium were detected in the circulation of UC patients that were cross-reactive with *Escherichia coli* antigens, and the hypothesis was proposed that such immune cross-reactivity could represent a form of autoimmunity relevant to IBD pathogenesis (Perlmann *et al.*, 1967). These initial reports were followed by a long series of studies demonstrating that both CD and UC patients possess antibodies against a range of potential autoantigens, including lymphocyte antigens (Korsmeyer *et al.*, 1974), cytoskeletal proteins (Mayet *et al.*, 1990), cardiolipin (Aichbichler *et al.*, 1999) and pancreatic proteins (Fricke *et al.*, 1999).

Additional evidence for the possible involvement of antibody-dependent pathophysiology in IBD was introduced by the demonstration of alterations of serum immunoglobulins and the presence of rheumatoid factor and anti-F(ab′)2 autoantibodies (MacDermott *et al.*, 1981; Pallone *et al.*, 1986), as well as circulating complement-fixing immune complexes in the serum of CD and UC patients (Doe *et al.*, 1973; Jewell and McLennan, 1973). Moreover, activated complement was located in the microvessels of IBD-involved mucosa (Halstensen *et al.*, 1989), and also in the gut epithelium in association with IgG1 antibodies (Halstensen *et al.*, 1993). Based on these observations, the question of whether IBD could be a true, autoantibody-dependent autoimmune disorder was raised (Thayer, 1976; Snook, 1990), even though convincing verification for the existence of organ-specific pathogenic autoantibodies had not been obtained.

### Autoantibodies to Intestinal Epithelial Antigens

The search for gut-specific autoantibodies has been relatively limited in IBD, and only two lines of investigation have yielded reasonable evidence suggesting that true autoantibodies may contribute to IBD pathogenesis.

Goblet cell glycoproteins, termed epithelial cell-associated components (ECAC), were initially identified in the rat intestine and later in the human intestine (Roche *et al.*, 1981; Aronson *et al.*, 1983), and the detection of ECAC-specific reactivity by circulating mononuclear cells and sera from CD and UC patients suggested that autosensitization to these protein had occurred in subjects with IBD (Aronson *et al.*, 1983). Antigen-specific reactivity against ECAC was later demonstrated using mononuclear cells isolated from CD and UC mucosa (Roche *et al.*, 1985). These mucosal cells were shown to be CD3+ lymphocytes, which induced antibody-mediated cytotoxicity for small or large bowel-derived ECAC but not control antigens (Roche *et al.*, 1985). These findings, including the localization of the antigen(s) to goblet cells and apparent antigen-specific immune reactivity, were recapitulated in the cotton top tamarin, a spontaneous model of IBD resembling UC (Winter *et al.*, 1989). These observations have not been followed by further biochemical characterization of the putative autoantigens or the mechanisms of autosensitization, and the importance of ECAC as a pathogenic autoantigen in IBD remains undefined.

Another series of studies has pursued a different potential autoantigen which, unlike ECAC, appears to be large bowel- and disease-specific. IgG antibodies eluted from the colonic mucosa of UC patients, but not from patients with CD or other colonic inflammatory conditions, were found to recognize a 40 kDa protein present exclusively in large bowel tissue, suggesting the possibility that such protein could represent an autoantigen mediating an antibody-mediated response in UC patients (Takahashi and Das, 1985). The generation of monoclonal antibodies against the 40 kDa protein permitted to define its exclusive localization to epithelial cells of the human large bowel, and not upper or small bowel, liver, pancreas or non gastrointestinal tissues (Das *et al.*, 1987). Interestingly, IgG1 and activated complement were found to colocalize with the 40 kDa on colonocytes of UC but not CD patients (Halstensen *et al.*, 1993). Follow up studies showed that the epitope recognized by the monoclonal antibodies in human colon was selectively present in skin, eye, joint and biliary epithelium (Das *et al.*, 1990; Bhagat and Das, 1994; Mandal *et al.*, 1994). This localization is extremely intriguing because those tissues match exactly the sites where the major extra-intestinal manifestations of IBD

occur, and led to the speculation that autoantibodies or immune cells sensitized to the 40 kDa colonic protein could cross-react with the same epitope in other locations, trigger local pathology and explain the extraintestinal manifestations of IBD (Das, 1999). Further investigation revealed that lamina propria mononuclear cells isolated from UC mucosa spontaneously produced IgG1 against the putative autoantigen (Biancone *et al.*, 1995), that has now been identified as a cytoskeletal protein of the tropomyosin family, specifically human tropomyosin fraction 5 (huTM5) (Das *et al.*, 1993; Geng *et al.*, 1998). Studies in animal models of IBD have yet to be performed to further substantiate the disease-causing potential of huTM5, and studies are under way to understand how this intracellular protein may be transported to the colonocyte surface to act as a target self-antigen. Thus, as is the case of ECAC, the true significance of huTM5 as a pathogenic autoantigen and the related mechanisms of colon inflammation are still uncertain.

### pANCA and ASCA

In addition to epithelial-cell autoantibodies, two other types of antibodies were commonly detected in IBD patients: antineutrophil cytoplasmic antibodies (ANCA) and anti-*Saccharomyces cerevisiae* antibodies (ASCA), which are relatively specific for UC and CD, respectively.

In the original report, sera from the majority of patients with UC were found to contain antibodies recognizing an antigen(s) with a nongranular perinuclear (p) distribution in neutrophils (Saxon *et al.*, 1990). The UC-associated pANCA were distinct from the ANCA found in Wegener's granulomatosis or crescent glomerulonephritis, and did not react with myeloperoxidase, suggesting that they may "UC-specific". In subsequent studies, the levels of pANCA in UC were consistently found to be significantly higher than those of patients with CD, infectious colitides, irritable bowel syndrome and miscellaneous diarrheal diseases, with a sensitivity of pANCA for UC of around 60% with a specificity of 80–90% (Duerr *et al.*, 1991). These values were based on mostly white North American patients with UC, but are lower in other European or Asian populations (Sugi *et al.*, 1999). Of interest, pANCA are present in the circulation of some animal models of experimental colitis, such as the interleukin (IL)-10-deficient and the T-cell receptor (TCR) α-deficient mice (Mizoguchi *et al.*, 1997; Seibold *et al.*, 1998). The neutrophil antigen(s) recognized by pANCA is still a matter of dispute, but it is clearly different from that associated with vasculitis. However, the answer to the more fundamental question of whether these autoantibodies are pathogenic and can induce gut inflammation appears to be a negative one. In fact, levels of pANCA in UC are not correlated to disease activity or extent, do not affect neutrophil function, patients can have UC without developing pANCA, and pANCA also occur in unaffected relatives of UC patients (Shanahan, 1994). However, pANCA may still be useful as serological markers of disease, possible markers of genetic susceptibility, or markers for disease heterogeneity.

At the same time that pANCA emerged as UC-associated autoantibodies, an association of CD with ASCA was also being investigated. Levels of serum antibodies against multiple strains of *S. cerevisiae* (baker's and brewer's yeast) were found to be significantly elevated in the serum of CD patients, but not serum of UC patients (McKenzie *et al.*, 1990; Giaffer *et al.*, 1992). This was initially proposed as potentially indicating hypersensitivity to dietary antigens in CD. This hypothesis has not been pursued to any significant extent and is still a theoretical possibility, but it is well established that ASCA recognize mannose sequences in the cell wall mannan of *S. cerevisiae*, defining ASCA as a non autoantigen in IBD. Although not totally specific for CD, as ASCA can be detected in serum of some celiac disease patients (Giaffer *et al.*, 1992), the lack of antibodies in the circulation of UC patients suggested that the combined measurement of pANCA and ASCA could help in the differential diagnosis of the two forms of IBD, a typically challenging situation for the practicing gastroenterologist. Two studies, one performed with adult and the other with pediatric subjects in which sera from UC and CD patients were assessed for both pANCA and ASCA, showed that the combination of a positive pANCA and a negative ASCA was highly specific (95–100%) for UC, whereas the combination of a negative pANCA and a positive ASCA was highly specific (95–100%) for CD (Ruemelle *et al.*, 1998; Quinton *et al.*, 1998). These results have been replicated in multiple reports, but unfortunately all studies agree that the sensitivity of the combined pANCA plus ASCA test is still too low (around 50–60%) to be useful as general screening tool.

### IMMUNE-MEDIATED EVENTS IN IBD

### Immune Abnormalities in IBD

Taking into consideration all the information discussed so far, it is apparent that data favoring classical autoimmune pathogenic mechanisms, like antigen-specific autoreactive T- or B-cells, are scant and not necessarily robust in IBD. On the other hand, there is an overwhelming amount of data proving that abnormalities of the mucosal and systemic immune systems are intimately involved in the pathogenesis of both CD and UC. As indicated earlier, these abnormalities are almost certainly secondary, and occur in the context of other *sine qua non* conditioning factors, which include genetic predisposition, environmental changes, and the intestinal flora (Fiocchi, 1998; Podolsky, 2002). Most of the immune abnormalities described in the last two decades of investigation have been focused on mucosal immune events, particularly searching for imbalances or skewing of the Th1/Th2 paradigm (Neurath *et al.*, 2002). Although the downstream events of mucosal inflammation are nonspecific and predictably characterized by the production of high levels of cytokines, growth factors, free radicals and matrix-degrading enzymes, a clear dichotomy exists in regard to dysregulated upstream immune events in IBD (Monteleone *et al.*, 2002). The transmural inflammation characteristic of CD is associated with a typical Th1 response dominated by high IL-12, IL-18 and interferon (IFN)-$\gamma$ production (Monteleone *et al.*, 1997), whereas UC has been very recently defined as an atypical Th2 response mediated by CD1d-restricted NK T-cells that produce high levels of IL-13 (Fuss *et al.*, 2004). Thus, the immune aberrations underlying the two main forms of IBD are clearly diverse. This conclusion, however, does not preclude the possibility that triggering phenomena or sensitizing agents are similar, if not the same. In fact, accumulating evidence derived from genetic, microbial and immunological observations strongly suggests that the normal indigenous flora of the intestine may be at the center of pathogenic events in IBD, a major point that will be the focus of the following discussion.

### The Intestinal Flora as a Tolerizing "Self-antigen"

Bacteria are usually regarded as microorganisms derived from the surrounding environment that have the capacity to invade the host and eventually cause disease. In reality, the body harbors huge quantities of non-pathogenic "friendly" bacteria, the vast majority of which are located within the intestinal lumen. The gut contains at least 400 different species of mostly anaerobic bacteria, and the number of microbial cells in the lumen is approximately ten times greater than that of the eukaryotic cells in the human body. This enormous amount of enteric microbes constantly and intimately interacts with the host and confers benefits that are essential to health and survival (Guarnier and Malagelada, 2003). DNA microarray analysis of the effect of colonization of germ-free mice with the *Bacteroides thetaiotaomicron*, a dominant component of normal human and murine flora, shows that this bacterium modulates expression of genes involved in intestinal nutrient absorption, mucosal barrier function, xenobiotics metabolism, angiogenesis and postnatal maturation (Hooper *et al.*, 2001). Not only the intestinal flora provides metabolic, trophic and protective functions to the host, but is also critical to the development of normal immunity by affecting the timing, maturation and composition of the mucosal immune system (Cebra *et al.*, 1998). Therefore, when intestinal colonization starts right after birth, the immune system learns to recognize bacteria and discriminate between commensal and potential pathogenic microorganisms. This is accomplished primarily by the innate immune system through toll-like receptors (TLR), surface structures that function as mammalian pattern-recognition receptors and are essential for the detection of microbial components (Takeda *et al.*, 2003). In addition to innate immunity, adaptive immunity to enteric bacteria will also develop.

In order to survive and thrive in a potentially hostile environment, bacteria have developed strategies that overcome the host's innate and adaptive immune responses, which include evasion of immune recognition, resistance to antibacterial epithelial effect molecules, escape from phagocyte responses, interference with antigen presentation, and inhibition of T- and B-cell functions (Hornef *et al.*, 2002). As a counterpart, the local mucosal immune system must learn to recognize the commensal enteric flora without destroying it. When genetically normal mice kept in a germ-free environment are given food mixed with fecal pellets from congenic mice containing normal flora, they develop an acute self-limited colitis which resolves quickly and is followed by the induction of a variety of small and large intestine-specific genes (Ogawa *et al.*, 2000). This suggests that when bacteria are first introduced in the gut they function as foreign antigens and induce an inflammatory response. Later, once recognized and found to be non pathogenic, the local immune cells no longer attack the bacteria with the intent to destroy them, and both enter a state a peaceful symbiotic co-existence. This can be viewed as similar to what happens when the immune system encounters a self-antigen that is a normal body component and toward which has already developed tolerance. Thus, a fundamental aspect of commensal host-bacterial relationships in the gut is the development and maintenance of immune tolerance to the gut enteric flora (Hooper and Gordon, 2001). Therefore, although the commensal gut flora technically is a foreign antigen, under physiological circumstances it behaves as a "self-antigen", and is perceived as such by the host immune system. Based on this concept, if tolerance to the gut commensal flora is lost, this becomes a pathogenic event that may lead to a state of chronic intestinal inflammation.

### Failure to Regulate Reactivity to the Gut Flora: A Central Event in IBD Pathogenesis?

There is mounting evidence that IBD represents a condition where the normal homeostatic balance between the enteric flora and mucosal immunity is lost, resulting in the chronic inflammatory response that typifies CD and UC. Three scenarios can be envisioned that might explain this outcome: *first*, the quantitative or qualitative composition of the flora has changed; *second*, the immune response towards the normal gut flora is abnormal; *third*, the regulatory mechanisms that control flora-host interaction have gone away.

### *The Intestinal Flora in IBD*

The complexity of the human gut flora has long represented a major obstacle to the full characterization of the innumerous microorganisms scattered all along the gastrointestinal tract. Nevertheless, the major groups of aerobic and anaerobic bacteria are known, and their progressive numerical increase from the upper to the lower bowel is well-known (Guarnier and Malagelada, 2003). The complexity of the gut flora rapidly increases after birth, and by the age of one year its composition resembles that of an adult person (Favier *et al.*, 2002). There are also observations indicating that, even though gut bacteria are relatively stable throughout the life of an individual, each person has a customized flora and that variations in bacterial composition occur depending on feeding (breast milk vs. bottle), different environments, and use of antibiotics, prebiotics and probiotics.

Recent reports have indicated that a relationship exists between the composition of the intestinal microflora and the propensity to suffer from allergic inflammation (Kirjavainen *et al.*, 2002), and there are differences in gut microbiota between infants who will and those who will not develop allergies even before the appearance of any clinical manifestations of atopy (Bjorksten *et al.*, 2001). The same may be true for IBD, as suggested by prospective studies showing that children with an abnormal flora have a greater chance of developing CD than children with a normal flora (VandeMerwe *et al.*, 1988). These results are compatible with two important concepts previously introduced: that the gut flora participates of the education of systemic and mucosal immunity, and that an altered flora may lead to an aberrant immune response. There are few studies that have analyzed the microflora of adults with established IBD, but it appears that both quantitative and qualitative differences are present in both CD and UC patients. In patients with UC there is a significant decrease in the number of anaerobic bacteria, anaerobic gram-negatives and lactobacilli (Fabia *et al.*, 1993), while in CD patients there is a significant increase in enterobacteria independent of the clinical activity of disease (Seksik *et al.*, 2003). In addition, the total number of bacteria found in the colonic mucus of IBD patients is significantly increased compared to controls (Schultsz *et al.*, 1999), and this number increases with the severity of mucosal inflammation (Swidsinski *et al.*, 2002). Together, these observations support the notion that alteration of the gut commensal flora may change its immune recognition from a "self-antigen" to a new or foreign antigen towards which the local immune system may mount a response which is manifested as IBD.

### *Enhanced Reactivity to Bacterial Antigens in IBD*

The possibility that patients with IBD develop an aberrant immune response against components of the gut flora has been under consideration for a long time. A number of reports show that CD and UC patients have increased titers of antibodies against *E. coli*, aerobes, anaerobes and even enteric bacterial pathogens, and that these antibodies are of both systemic and mucosal origin (Monteiro *et al.*, 1971; Tabaqchali *et al.*, 1978; Blaser *et al.*, 1984; Macpherson *et al.*, 1996). The previously discussed ASCA in CD patients probably reflect the same type of broad anti-microbial reactivity associated with IBD (McKenzie *et al.*, 1990; Giaffer *et al.*, 1992). More recently, novel gut bacterial antigens have been reported in association with IBD, including the *Pseudomonas fluorescens*-associated sequence I2, the outer membrane porin C of *E. coli* (OmpC), and bacterial flagellins, towards which CD patients develop significantly higher antibody titers than UC or control subjects (Sutton *et al.*, 2000; Landers *et al.*, 2002; Lodes *et al.*, 2004). Interestingly, it has been suggested that the higher the antibody responses against bacteria and more numerous the antigens against which they react, the more likely it is that CD patients will experience a more severe clinical course, as indicated by a greater frequency of strictures, perforations, and surgical interventions (Mow *et al.*, 2004). If confirmed, this observation would indicate that the greater the level of sensitization to enteric bacteria, the stronger and more damaging the ensuing mucosal immune response might be.

Fewer data are available on the cell-mediated immune response to bacterial antigens in IBD. The existing reports also indicate that there is an enhanced T-cell reactivity against microbial antigens of enteric and non enteric origin in IBD, particularly in CD patients, at both the systemic and intestinal mucosa level (Bull and Ignaczak, 1973; Pirzer *et al.*, 1991; Young *et al.*, 1994; Duchmann *et al.*, 1999). Supporting evidence for sensitization to bacterial antigens in IBD can also be found in animal models of experimental colitis, where CD4+ T-cells reactive against enteric flora are detected in both the spleen and the colon, and they can transfer IBD in adoptive transfer experiments in SCID mice (Cong *et al.*, 1998; Wirtz *et al.*, 1999). In addition, it is worth noticing that some enteric antigens like I2, to which enhanced humoral immune responses are found in IBD, also function as superantigens (Dalwadi *et al.*, 2001), and non enteric superantigens like *Staphylococcus aureus* enterotoxin B, or enteric superantigens like *Yersinia pseudotuberculosis* can activate T-cells and elicit or aggravate gut inflammation of which they are not the causative agents (Lu *et al.*, 2003).

### *Abnormal Regulation of Anti-bacterial Reactivity in IBD: Loss of Tolerance to the Enteric Flora*

While the above-mentioned studies certainly indicate that abnormal immune reactivity to bacteria is intimately associated with IBD pathogenesis, the essential role of the luminal microbiota in triggering or maintaining IBD was only confirmed with the use of animal models in which the presence, quantity and type of the flora could be experimentally controlled and manipulated. The report that a germ-free condition prevents development of gut inflammation in HLA-B27 transgenic rats was the first to bring attention to the fact that experimental IBD is dependent on the presence of intestinal bacteria (Taurog *et al.*, 1994). This seminal observation was followed by others showing that the luminal bacterial load and its composition together determine the degree of inflammation, and different strains display intrinsically dissimilar proinflammatory capacities, that vary from potent to moderate to absent depending on the genetic background of the host (Rath *et al.*, 1999a,b). The observation that IBD fails to develop in the absence of enteric flora has now been confirmed in numerous animal models of IBD, and has lead to the widely accepted paradigm “no bacteria, no colitis”.

If the commensal gut flora is required to develop IBD, and an inappropriately strong immune response against it is a key mechanism responsible for the development of chronic intestinal inflammation, how does such damaging immune response develop? One possibility, already discussed, is that the antigenic properties of the flora have changed and an immune response is mounted to what previously was a “self-antigen” towards which tolerance had been established. The reverse possibility is that the flora is not significantly altered in IBD, but the normal state of tolerance to luminal bacterial antigens has been lost (Khoo *et al.*, 1997). In regard to the second possibility, a classical study revealed that human peripheral blood and mucosal mononuclear cells fail to proliferate when exposed to bacteria from autologous intestine, but do so after exposure to bacteria from heterologous intestine, indicating that tolerance to the autologous gut microbiota normally exists (Duchmann *et al.*, 1995). In contrast, mucosal immune cells from active CD or UC patients vigorously proliferate after co-culture with bacteria from the autologous intestine, indicating that tolerance is lost in IBD. Similar findings were seen in a hapten-induced model of experimental IBD (Duchmann *et al.*, 1996). Whether loss of tolerance in IBD is broadly directed to the whole flora or only some components of it is not clear yet, but the presence of quantitatively and qualitatively different antibodies to multiple microbial antigens in various patients subsets suggests that a selected, rather than a global, loss of tolerance occurs in IBD (Landers *et al.*, 2002). The extent and severity of this loss of tolerance is still being defined, as it has been recently demonstrated that loss of tolerance in IBD patients is not exclusive for bacterial antigens and occurs also to orally administered soluble proteins (Kraus *et al.*, 2004).

### *Potential Mechanisms Contributing to the Loss of Tolerance in IBD*

The exact cause for the loss of tolerance in IBD is not known. This is an extremely active area of investigation and specific mechanisms are under consideration, some of which will be briefly discussed.

Loss of tolerance can be viewed as an inadequate function of immunoregulatory cells. This rather traditional view has gained considerable strength with the identification of specific subsets of immunoregulatory cells, among which inducible type 1 T regulatory cells (Tr1) and spontaneously occurring *FOXP3*+CD4+CD25+ regulatory cells figure prominently (Roncarolo *et al.*, 2001; Bach, 2003). There is substantial evidence, primarily from animal models of IBD, that regulatory T cell are involved in the control of intestinal inflammation (Toms and Powrie, 2001). CD4+, IL-10-secreting Tr1 cells suppress antigen-specific immune responses and actively prevents experimental colitis (Groux *et al.*, 1997), and CD4+ CD25+ regulatory T-cells can cure established murine colitis through mechanisms involving cell-to-cell contact as well as IL-10 and transforming growth factor β1 (Mottet *et al.*, 2003). Several laboratories are presently investigating the function of immunoregulatory T-cells in humans with IBD, and defects in the number or activity of these cells are likely to emerge in CD or UC patients. However, even after detection, the true significance of immunoregulatory defects in IBD will be difficult to interpret and fit into IBD pathogenesis because of the intricate and mutual regulatory networks between regulatory T-cells and other immune cells, and the complex nature of bacterial antigen recognition. For instance, microbial activation of TLR on dendritic cells (DC) can block the suppressor effect of CD4+ CD25+ regulatory cells and allow a pathogen-specific immune response to occur (Casare and Medzhitov, 2003). On other hand, CD4+CD25+ T-cells can restrain the maturation and antigen-presenting function of DC (Misra *et al.*, 2004), which, as mentioned below, will affect their capacity of recognize and respond to bacterial stimuli.

Dendritic cells are a heterogeneous population of bone marrow-derived antigen-presenting cells that influence essentially all aspects of innate and acquired immunity (Liu, 2001). They sense the surrounding microbial environment through TLR, and signaling through different TLR generates distinct biological responses, which vary from excitatory to suppressive. In the intestine DC are found scattered in all lymphoid compartments, displaying distinct properties and functions (Staag *et al.*, 2003), and can penetrate the space in between epithelial cells and sample lumenal bacteria that they subsequently present to immune cells in the mucosa (Rescigno *et al.*, 2001). It is now generally believed that DC are critical to the balance between tolerance and active immunity and, because intestinal DC appear to be excessively activated in IBD (Staag *et al.*, 2003), it is possible that their function is skewed towards active immunity rather than tolerance.

Due to the persistent and massive presence of the commensal flora, intestinal epithelial cells may have adapted by developing mechanisms that avoid activation by TLR-dependent microbial signals with a pro-inflammatory potential. In support of this hypothesis recent reports show that human intestinal epithelial cells are broadly unresponsive to ligation of TLR2, which recognizes Gram-positive cell wall components such as peptidoglycan and certain lipoproteins (Melmed *et al.*, 2003). In addition, while short term stimulation with bacterial lipolysaccharide or lipoteichoic acid activates pro-inflammatory cascades in epithelial cells, prolonged stimulation—mimicking what occurs in the intestinal milieu—results in state of prolonged hyporesponsiveness associated with downregulation of TLR surface expression (Otte *et al.*, 2004). Changes in bacterial flora composition or alteration of epithelial cell surface receptor expression could bypass these control mechanisms that prevent excessive activation by bacteria, and result in sustained pro-inflammatory signals blocking the development of tolerance.

A genetic defect could be at the root of the issue of broken tolerance in IBD. This, until recently, far-fetched conjecture has suddenly become a realistic possibility with the discovery of the association of CD with mutations of the *NOD2/CARD15* gene on chromosome 16, the so-called *IBD1* locus (Hugot *et al.*, 2001; Ogura *et al.*, 2001). The gene product of *NOD2/CARD15* is a cytosolic protein that activates NF-κB following intracellular stimulation by bacterial products, and NOD2/CARD15 normally functions as a general sensor of peptidoglycan through the recognition of muramyl dipeptide, the minimal bioactive peptidoglycan motif common to all bacteria (Girardin *et al.*, 2003). In view of the well established connection between recognition of bacterial products and CD pathogenesis, variants of the NOD2/CARD15 protein with impaired recognition capacity and altered downstream signaling acquire critical importance in the development and control of gut inflammation. At first sight, given its NF-κB activating function, it is difficult to reconcile the paradox that an apparent "loss of function" of NOD2/CARD15 results in inflammation. One possibility is that the host compensates this loss of function with an excessive or protracted activation of adaptive immunity, and the other possibility is that the NOD2 protein does not necessarily behave a pro-inflammatory molecule (Girardin *et al.*, 2003). Two recent reports lend support to the second possibility. Normal NOD2-expressing-epithelial cells are resistant to invasion by *Salmonella typhimurium*, while those carrying the CD mutations of NOD2 are unable to constrain bacterial growth, suggesting that NOD2/CARD15 is a component of innate immunity responses to luminal bacteria acting as an anti-bacterial factor (Hisamatsu *et al.*, 2003). Moreover, *NOD2*-deficient mice or carrying a CD-like *CARD15* mutation exhibit increased TLR2-mediated activation of NF-κB and excessive Th1 responses which may trigger inflammation (Watanabe *et al.*, 2004).

Finally, infections can break T-cell tolerance (Rocken *et al.*, 1992), although epidemiological or clinical evidence that infections precede the appearance of IBD is largely anecdotal. Nevertheless, the enormous bacterial load of the intestinal lumen may itself play an important role in breaking tolerance. In fact, recent studies show that sustained exposure to bacterial antigen induces down-regulation of the TCR ζ chain and impaired T-cell function (Bronstein-Sitton *et al.*, 2003), and activation of

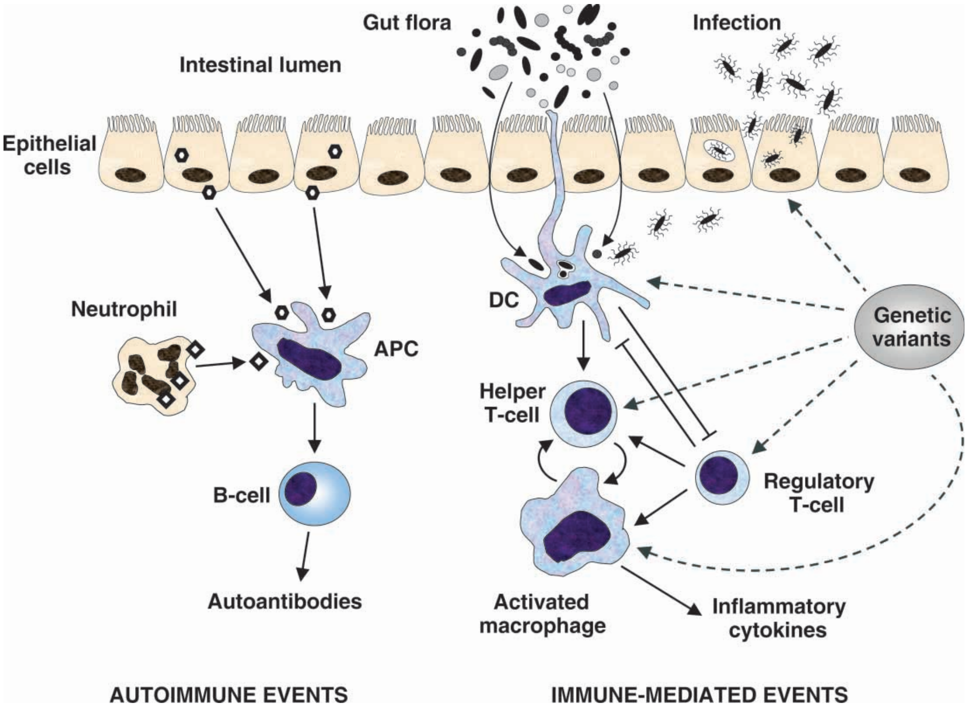

FIGURE 1 Schematic diagram of autoimmune and immune-mediated events in IBD pathogenesis. *Left*: Self-antigens derived from intestinal epithelial cells (hexagons), neutrophils (diamonds), and other host cells are internalized and processed by antigen-presenting cells (APC), and presented to B-cells which produce autoantibodies such as epithelial cell-associated components (ECAC), pANCA, lymphocytotoxic antibodies, anti-pancreas antibodies, etc. *Right*: Loss of tolerance to the commensal autologous flora results in an enhanced reactivity against gut bacterial antigens and the inappropriate activation of effector CD4+ helper T-cells which induce macrophage activation and production of pro-inflammatory cytokines. Possible causes for the loss of tolerance include infections, excessive dendritic cell (DC) stimulation by the gut flora, inadequate regulatory T-cell function, or genetic factors, such as the CD-associated *NOD2/CARD15* variants, that affect both epithelial and immune cell function.

antigen-presenting cells by microbial products via TLR breaks self tolerance and can induce autoimmune disease (Waldner *et al.*, 2004).

### CONCLUSIONS

Based on the available information, it seems fair to conclude that immune-mediated appear to be more important than autoimmune phenomena as overall pathogenic mechanisms of IBD. It seems also fair to conclude that some difference probably exists in regard to the relative contribution of autoimmune vs. immune-mediated phenomena in each form of IBD, since there is reasonable evidence for a role of autoreactivity against colonic epithelial cells in UC, whereas immune reactivity against intestinal flora is the prominent feature of CD (Fig. 1). However, simply addressing the contribution of the immune system to IBD pathogenesis, no matter in how many exquisite details, is unlikely to provide answers to the fundamental question of why these chronic inflammatory disorders have appeared in the last century and their prevalence and incidence continue to raise worldwide, affecting now populations where CD and UC were essentially unknown until a few decades ago. All chronic inflammatory diseases of unknown origin incorporate, in addition to immune dysregulation, genetic predisposition and environmental factors in their mechanisms of emergence (Ermann and Fatham, 2001). Unfortunately, the contribution of genes to disease development is still not amenable to therapeutic intervention, and returning our increasingly "clean" environment back to a "dirty" one, as proposed by the hygiene hypothesis (Bach, 2002), is not practically feasible. Thus, even though an immunotherapeutic approach to IBD addresses the mechanisms rather the cause of disease, the continued investigation of autoimmune and immune-mediated phenomena in IBD is the best hope to gain a therapeutic handling on these devastating conditions, but this must be done in parallel with a better understanding of the interactions, both symbiotic and pathologic, that occur between mucosal immunity and the commensal enteric flora.

### *References*

Aichbichler, B.W., Petritsch, W., Reicht, G.A., Wenzel, H.H., Eherer, A.J., Hinterleitner, T.A., *et al.* (1999) "Anti-cardiolipin antibodies in patients with inflammatory bowel disease", *Dig. Dis. Sci.* **44**, 852–856.

Aronson, R.A., Cook, S.L. and Roche, J.K. (1983) "Sensitization to epithelial antigens in chronic mucosal inflammatory disease. I. Purification, characterization, and immune reactivity of murine epithelial cell-associated components (ECAC)", *J. Immunol.* **131**, 2796–2804.

Bach, J-F. (2003) "Regulatory cells under scrutiny", *Nat. Rev.* **3**, 189–198.

Bach, J-F. (2002) "The effect of infections an susceptibility to autoimmune and allergic diseases", *N. Engl. J. Med.* **347**, 911–920.

Bhagat, S. and Das, K.M. (1994) "A shared and unique epitope in the human colon, eye and joint detected by a monoclonal antibody", *Gastroenterology* **107**, 103–108.

Biancone, L., Mandal, A., Yang, H., Dasgupta, T., Paoluzi, A.O., Marcheggiano, A., *et al.* (1995) "Production of immunoglobulin G and G1 antibodies to cytoskeletal protein by lamina prorpia cells in ulcerative colitis", *Gastroenterology* **109**, 3–12.

Bjorksten, B., Sepp, E., Julge, K., Voor, T. and Mikelsaar, M. (2001) "Allergy development and the intestinal microflora during the first years of life", *J. Allergy Clin. Immunol.* **108**, 516–520.

Blaser, M.J., Miller, R.A., Lacher, J. and Singleton, J.W. (1984) "Patients with active Crohn's disease have elevated serum antibodies to antigens of seven enteric bacterial pathogens", *Gastroenterology* **87**, 888–894.

Bouma, G. and Strober, W. (2003) "The immunological and genetic basis of inflammatory bowel disease", *Nat. Rev. Immunol* **3**, 521–533.

Broberger, O. and Perlmann, P. (1959) "Autoantibodies in human ulcerative colitis", *J. Exp. Med.* **110**, 657–674.

Bronstein-Sitton, N., Cohen-Daniel, L., Vakin, I., Ezernitchi, A.V., Leshen, B., Halabi, H., *et al.* (2003) "Sustained exposure to bacterial antigens induces interferon-$\gamma$-dependent T cell receptor $\eta$ down-regulation and impaired T cell function", *Nat. Immunol.* **4**, 957–964.

Bull, D.M. and Ignaczak, T.F. (1973) "Enterobacterial common antigen-induced lymphocyte reactivity in inflammatory bowel disease", *Gastroenterology* **64**, 43–50.

Casare, C. and Medzhitov, R. (2003) "Toll pathway-dependent blockade of CD4+ CD25+ T cell-mediated suppression by dendritic cells", *Science* **299**, 1033–1036.

Cebra, J.J., Periwal, S.B., Lee, G. and Shroff, K.E. (1998) "Development and maintenance of the gut-associated lymphoid tissue (GALT): the roles of enteric bacteria and viruses", *Dev. Immunol.* **6**, 13–18.

Cong, B.Y., Brandwein, S.L., McCabe, R.P., Lazenby, A., Birkenmeier, E.H., Sundberg, J.P., *et al.* (1998) "CD4+ T cells reactive to enteric bacterial antigens in spontaneously colitic C3H/HeJBir mice: increased T helper cell type 1 response and ability to transfer disease", *J. Exp. Med.* **187**, 855–864.

Dalwadi, H., Wei, B., Kronenberg, M., Sutton, C.L. and Braun, J. (2001) "The Crohn's disease-associated bacterial protein I2 is a novel enteric T cell superantigen", *Immunity* **15**, 149–158.

Das, K.M., Sakamaki, S., Vecchi, M. and Diamond, B. (1987) "The production and characterization of monoclonal antibodies to a human colonic antigen associated with ulcerative colitis: cellular localization of the antigen by using the monoclonal antibody", *J. Immunol.* **139**, 77–84.

Das, K.M., Vecchi, M. and Sakamaki, S. (1990) "A shared and unique epitope(s) on human colon, skin, and biliary epithelium detected by a monoclonal antibody", *Gastroenterology* **98**, 464–469.

Das, K.M. (1999) "Relationship of extraintestinal involvements in inflammatory bowel disease. New insights into autopimmune pathogenesis", *Dig. Dis. Sci.* **44**, 1–13.

Das, K.M., Dasgupta, A., Mandal, A. and Geng, X. (1993) "Autoimmunity to cytoskeletal protein tropomyosin. A clue to the pathogenetic mechanisms for ulcerative colitis", *J. Immunol.* **150**, 2487–2493.

Doe, W.F., Booth, C.C. and Brown, D.L. (1973) "Evidence for complement-binding immune complexes in adult coeliac disease, Crohn's disease and ulcerative colitis", *Lancet* **24**, 402–403.

Duchmann, R., Kaiser, I., Hermann, E., Mayet, W., Ewe, K. and Meyer zum Buschenfelde, K.H. (1995) "Tolerance exists towards resident intestinal flora but it is broken in active inflammatory bowel disease (IBD)", *Clin. Exp. Immunol.* **102**, 448–455.

Duchmann, R., Schmitt, E., Knolle, P., Meyer zum Buschenfelde, K.H. and Neurath, M. (1996) "Tolerance towards resident intestinal flora in mice is abrogated in experimental colitis and restored by treatment with interlukin-10 or antibodies to interleukin-12", *Eur. J. Immunol.* **26**, 934–938.

Duchmann, R., May, E., Heike, M., Knolle, P., Neurath, M. and Meyer zum Buschenfelde, K.H. (1999) "T cell specificity and cross reactivity towards enterobacteria, Bacteroides, Bifidobacterium, and antigens from resident intestinal flora in humans", *Gut* **44**, 812–818.

Duerr, R.H., Targan, S.R., Landers, C.J., Sutherland, L.R. and Shanahan, F. (1991) "Anti-neutrophil cytoplasmic antibodies in ulcerative colitis. Comparison with other colitides/diarrheal diseases", *Gastroenterology* **100**, 1590–1596.

Ermann, J. and Fatham, C.G. (2001) "Autoimmune diseases: genes, bugs and failed regulation", *Nat. Immunol.* **2**, 759–761.

Fabia, R., ArRajab, A., Andersson, M-L., Willen, R., Jeppsson, B., Molin, G. and Bengmark, S. (1993) "Impairement of bacterial flora in human ulcerative colitis and experimental colitis in the rat", *Digestion* **54**, 248–255.

Favier, C.F., Vaughan, E.E., DeVos, W.M. and Akkermans, A.D.L. (2002) "Molecular monitoring of succession of bacteria communities in human neonates", *Appl. Environ. Microbiol.* **68**, 219–226.

Fiocchi, C. (1998) "Inflammatory bowel disease: etiology and pathogenesis", *Gastroenterology* **115**, 182–205.

Fricke, H., Birkhofer, A., Folwaczny, C., Meister, W. and Scriba, P.C. (1999) "Characterization of antigens from the human exocrine pancreatic tissue (Pag) relevant as target antigens for autoantibodies in Crohn's disease", *Eur. J. Clin. Investig.* **29**, 41–45.

Fuss, I.J., Heller, F., Boirivant, M., Leon, F., Yoshida, M., Fichtner-Feigi, S., *et al.* (2004) "Nonclassical CD1d-restricted NK T cells that produce IL-13 characterize an atypical Th2 response in ulcerative colitis", *J. Clin. Investig.* **113**, 1490–1497.

Geng, X., Biancone, L., Dai, H.H., Lin, J.J.C., Yoshizaki, N., Dasgupta, A., *et al.* (1998) "Tropomyosin isoform in intestinal mucosa: production of autoantibodies to tropomyosin isoforms in ulcerative colitis", *Gastroenterology* **114**, 912–922.

Giaffer, M.H., Clark, A. and Holdsworth, C.D. (1992) "Antibodies to Saccharomyce cerevisiae in patients with Crohn's disease and their possible pathogenic importance", *Gut* **33**, 1071–1075.

Girardin, S.E., Boneca, I.G., Viala, J., Chamaillard, M., Labigne, A., Thomas, G., *et al.* (2003) "Nod2 is a general sensor of peptidoglycan through muramyl dipeptide (MDP) detection", *J. Biol. Chem.* **278**, 8869–8872.

Girardin, S.E., Hugot, J.P. and Sansonetti, P.J. (2003) "Lessons from Nod2 studies: towards a link between Crohn's disease and bacterial sensing", *Trends Immunol.* **24**, 652–658.

Groux, H., O'Garra, A., Bigler, M., Rouleau, M., Antonenko, S., deVries, J.E., *et al.* (1997) "A CD4+ T-cell subset inhibits antigen-specific T-cell responses and prevents colitis", *Nature* **389**, 737–742.

Guarnier, F. and Malagelada, J.R. (2003) "Gut flora in health and disease", *Lancet* **361**, 512–519.

Halstensen, T.S., Mollnes, T.E. and Brandtzaeg, P. (1989) "Persistent complement activation in submucosal vessels of active inflammatory bowel disease: immunohistochemical evidence", *Gastroenterology* **97**, 10–19.

Halstensen, T.S., Das, K.M. and Brandtzaeg, P. (1993) "Epithelial deposits of immunoglobulin G1 and activated complement colocalise with the Mr 40 kDa putative autoantigen in ulcerative colitis", *Gut* **34**, 650–657.

Hisamatsu, T., Suzuki, M., Reinecker, H.C., Nadeau, W.J., McCormick, B.A. and Podolsky, D.K. (2003) "CARD15/NOD2 functions as an antibacterial factor in human intestinal epithelial cells", *Gastroenterology* **124**, 993–1000.

Hooper, L.V. and Gordon, J.I. (2001) "Commensal host-bacterial relationships in the gut", *Science* **292**, 1115–1118.

Hooper, L.V., Wong, M.H., Thelin, A., Hansson, L., Falk, P.G. and Gordon, J.I. (2001) "Molecular analysis of commensal host-microbial relationships in the intestine", *Science* **291**, 881–884.

Hornef, M.W., Wick, M.J., Rhen, M. and Normark, S. (2002) "Bacterial strategies for overcoming host innate and daptive immune responses", *Nat. Immunol.* **3**, 1033–1040.

Hugot, J.P., Chamaiilard, M., Zouali, H., Lesage, S., Cezard, J.P., Belaiche, J., *et al.* (2001) "Association of NOD2 leucine-rich repeat variants with susceptibility to Crohn's disease", *Nature* **411**, 599–603.

Jewell, D.P. and McLennan, I.C.M. (1973) "Circulating immune complexes in inflammatory bowel disease", *Clin. Exp. Immunol.* **14**, 219–226.

Khoo, U.Y., Proctor, I.E. and Macpherson, A.J.S. (1997) "CD4+ T cell down-regulation in human intestinal mucosa. Evidence for intestinal tolerance to luminal bacterial antigens", *J. Immunol.* **158**, 3626–3634.

Kirjavainen, P.V., Arvola, T., Dalminen, S.J. and Isolauri, E. (2002) "Aberrant composition of gut microbiota of allergic infants: a target of bifidobacterial therapy at weaning?", *Gut* **51**, 51–55.

Korsmeyer, S., Strickland, R.G., Wilson, I.D. and Williams, R.C. (1974) "Serum lymphocytotoxic and lymphocytophilic antibody activity in inflammatory bowel disease", *Gastroenterology* **67**, 578–583.

Kraus, T.A., Toy, L., Chan, L., Childs, J. and Mayer, L. (2004) "Failure to induce oral tolerance to a soluble protein in patients with inflammatory bowel disease", *Gastroenterology* **126**, 1771–1778.

Landers, C.J., Cohavy, O., Misra, R., Huiying, H., Lin, Y.C., Braun, J., *et al.* (2002) "Selected loss of tolerance evidenced by Crohn's disease-associated immune responses to auto- and microbial antigens", *Gastroenterology* **123**, 689–699.

Liu, Y.J. (2001) "Dendritic cell subsets and lineages, and their functions in innate and adaptive immunity", *Cell* **106**, 259–262.

Lodes, M.J., Cong, Y., Elson, C.O., Mohamath, R., Landers, C.J., Targan, S.R., *et al.* (2004) "Bacterial flagellin is a dominant antigen in Crohn disease", *J. Clin. Investig.* **113**, 1296–1306.

Lu, J., Wang, A., Ansari, S., Hershberg, R.M. and McKay, D.M. (2003) "Colonic bacterial superantigens evoke an inflammatory response and exaggerate disease in mice recovering from colitis", *Gastroenterology* **125**, 1785–1795.

MacDermott, R.P., Nash, G.S., Bertovich, M.J., Seiden, M.V., Bragdon, M.J. and Beale, M.G. (1981) "Alterations of IgM, IgG, and IgA synthesis and secretion by peripheral blood and intestinal mononuclear cells from patients with ulcerative colitis and Crohn's disease", *Gastroenterology* **81**, 844–852.

Macpherson, A., Khoo, U.Y., Forgacs, I., Philipott-Howard, J. and Bjarnarson, I. (1996) "Mucosal antibodies in inflammatory bowel disease are directed against intestinal bacteria", *Gut* **38**, 365–375.

Mandal, A., Dasgupta, A., Jeffers, L., Squillante, L., Hyder, S., Reddy, R., *et al.* (1994) "Autoantibodies in sclerosing cholangitis against a shared peptide in biliary and colon epithelium", *Gastroenterology* **106**, 185–192.

Marrack, P., Kappler, J. and Kotzin, B.L. (2001) "Autoimmune disease: why and where it occurs", *Nat. Med.* **7**, 899–905.

Mayet, W.J., Press, A.G., Hermann, E., Moll, R., Manns, M., Ewe, K. and MeyerzumBuschenfelde, K.H. (1990) "Antibodies to cytoskeletal proteins in patients with Crohn's disease", *Eur. J. Clin. Investig.* **20**, 516–524.

McKenzie, H., Main, J., Pennington, C.R. and Parratt, D. (1990) "Antibody to selected strains of *Saccharomyces cerevisiae* (baker's and brewer' yeast) and Candida albicans in Crohn's disease", *Gut* **31**, 536–538.

Melmed, G., Thomas, L.S., Lee, N., Tesfay, S.Y., Lukasek, K., Michelsen, K.S., *et al.* (2003) "Human intestinal epithelial cells are broadly unresponsive to toll-like receptor 2-dependent bacterial ligands: implications for host-microbial interactions in the gut", *J. Immunol.* **170**, 1406–1415.

Misra, N., Bayry, J., Lacroix-Desmazes, S., Kazatchkine, M.D. and Kaveri, S.V. (2004) "Human CD4+ CD25+ T cells restrain the maturation and antigen-presenting function of dentritic cells", *J. Immunol.* **172**, 4676–4680.

Mizoguchi, E., Mizoguchi, A., Chiba, C., Niles, J.L. and Bhan, A.K. (1997) "Antineutrophil cytoplasmic antibodies in T-cell receptor α-deficient mice with chronic colitis", *Gastroenterology* **113**, 1828–1835.

Monteiro, E., Fossey, J., Shiner, M., Drasar, B.S. and Allison, A.C. (1971) "Antibacterial antibodies in rectal and colonic mucosa in ulcerative colitis", *Lancet* **i**, 249–251.

Monteleone, G., Biancone, L., Marasco, R., Morrone, G., Marasco, O., Luzza, F., *et al.* (1997) "Interleukin 12 is expressed and actively released by Crohn's disease intestinal lamina propria mononuclear cells", *Gastroenterology* **112**, 1169–1178.

Monteleone, I., Vavassori, P., Biancone, L., Monteleone, G. and Pallone, F. (2002) "Immunoregulation in the gut: success and failures in human disease", *Gut* **50**(Suppl III), iii60–iii64.

Mottet, C., Uhlig, H.H. and Powrie, F. (2003) "Cure of colitis by $CD4^{+}CD25^{+}$ regulatory T cells", *J. Immunol.* **170**, 3939–3943.

Mow, W.S., Vasiliauskas, E.A., Lin, Y.C., Fleshner, P.R., Papadakis, K.A., Taylor, K.D., *et al.* (2004) "Association of antibody responses to microbial antigens and complications of small bowel Crohn's disease", *Gastroenterology* **126**, 414–424.

Neurath, M.F., Finotto, S. and Glimcher, L.H. (2002) "The role of Th1/Th2 polarization in mucosal immunity", *Nat. Med.* **8**, 567–573.

Ogawa, H., Fukushima, K., Sasaki, I. and Matsuno, S. (2000) "Identification of genes involved in mucosal defense and inflammation associated with normal enteric bacteria", *Am. J. Physiol.* **279**, G492–G499.

Ogura, Y., Bonen, D.K., Inohara, N., Nicolae, D.L., Chen, F.F., Ramos, R., *et al.* (2001) "A frameshift mutation in Nod2 associated with susceptibility to Crohn's disease", *Nature* **411**, 603–606.

Otte, J.M., Cario, E. and Podolsky, D.K. (2004) "Mechanisms of cross hyporesponsiveness to toll-like receptor bacterial ligands in intestinal epithlial cells", *Gastroenterology* **126**, 1054–1070.

Pallone, F., Matricardi, P.M., Squarcia, O., Fais, S., LeMoli, S., Boirivant, M., *et al.* (1986) "Raised serum levels of IgM-rheumatoid factor and anti-F(ab′)2 autoantibodies in patients with active inflammatory bowel disease", *J. Clin. Lab. Immunol.* **19**, 175–180.

Perlmann, P. and Broberger, O. (1963) "*In vitro* studies of ulcerative colitis. II. Cytotoxic action of white blood cells from patients on human fetal colon cells", *J. Exp. Med.* **117**, 717–733.

Perlmann, P., Hammarstrom, S., Lagercrantz, R. and Campbell, D. (1967) "Autoantibodies to colon in rats and human ulcerative colitis: cross reactivity with *Escherichia coli* O:14 antigen", *Proc. Soc. Exp. Biol. Med.* **125**, 975–980.

Pirzer, U., Schonhaar, A., Fleischer, B., Hermann, E. and Meyer zum Buschenfelde, K.H. (1991) "Reactivity of infiltrating T lymphocytes with microbial antigens in Crohn's disease", *Lancet* **338**, 1238–1239.

Podolsky, D.K. (2002) "Inflammatory bowel disease", *N. Engl. J. Med.* **347**, 417–429.

Quinton, J.F., Sendid, B., Reumaux, D., Duthilleul, P., Cortot, A., Grandbastien, B., *et al.* (1998) "Anti-Saccharomyces cerevisiae mannan antibodies combined with antineutrophil cytoplasmic autoantibodies in inflammatory bowel disease: prevalence and diagnostic role", *Gut* **42**, 788–791.

Rath, H.C., Ikeda, J.S., Linde, H.J., Scholmerich, J., Wilson, K.H. and Sartor, R.B. (1999a) "Varying cecal bacterial loads influences colitis and gastritis in HLA-B27 transgenic rats", *Gastroenterology* **116**, 310–319.

Rath, H.C., Wilson, K.H. and Sartor, R.B. (1999b) "Differential induction of colitis and gastritis in HLA-B27 trangenic rats selectively colonized with Bacteroides vulgatus or *Escherichia coli*", *Infect. Immunol.* **67**, 2969–2974.

Rescigno, M., Urbano, M., Valzasina, B., Francolini, M., Rotta, G., Bonasio, R., *et al.* (2001) "Dendritic cells express tight junction proteins and penetrate gut epithlial monolayers to sample bacteria", *Nat. Immunol.* **2**, 361–367.

Roche, J.K., Cook, S.L. and Day, E.D. (1981) "Goblet cell glycoprotein: an organ-specific antigen for the gut. Isolation, tissue localization and immune response", *Immunology* **44**, 799–810.

Roche, J.K., Fiocchi, C. and Youngman, K. (1985) "Sensitization to epithelial antigens in chronic mucosal inflammatory disease. Characterization of human intestinal mucosa-derived mononuclear cells reactive with purified epithelial cell-associated components *in vitro*", *J. Clin. Investig.* **75**, 522–530.

Rocken, M., Urban, J.F. and Shevach, E.M. (1992) "Infection breaks T-cell tolerance", *Nature* **359**, 79–82.

Roncarolo, M.G., Bacchetta, R., Bordignon, C., Narula, S. and Levings, M.K. (2001) "Type 1 regulatory cells", *Immunol. Rev.* **182**, 68–79.

Rose, N.R. and Bona, C. (1993) "Defining criteria for autoimmune diseases (Witebsky's postulates revisited)", *Immunol. Today* **14**, 426–430.

Ruemelle, F.M., Targan, S.R., Levy, G., Dubinsky, M., Braun, J. and Seidman, E.G. (1998) "Diagnostic accuracy of serological assays in pediatric inflammatory bowel disease", *Gastroenterology* **115**, 822–829.

Saxon, A., Shanahan, F., Landers, C., Ganz, T. and Targan, S. (1990) "A distinct subset of anti-neutrophil cytoplasmic antibodies is associated with inflammatory bowel disease", *J. Allergy Clin. Immunol.* **86**, 202–210.

Schultsz, C., VandenBerg, F.M., Kate, F.W.T., Tytgat, G.N.J. and Dankert, J. (1999) "The intestinal mucus layer from patients with inflammatory bowel disease harbors high numbers of bacteria compared with controls", *Gastroenterology* **117**, 1089–1097.

Seibold, F., Brandwein, S., Simpson, S., Terhorst, C. and Elson, C.O. (1998) "pANCA represents a cross-reactivity to enteric bacterial antigens", *J. Clin. Immunol.* **18**, 153–160.

Seksik, P., Rigottier-Gois, L., Gramet, G., Sutren, M., Pochart, P., Marteau, P., *et al.* (2003) "Alterations of the dominant faecal bacterial groups in patients with Crohn's disease of the colon", *Gut* **52**, 237–242.

Shanahan, F. (1994) "Neutrophil autoantibodies in inflammatory bowel disease: are they important?", *Gastroenterology* **107**, 586–589.

Snook, J. (1990) "Are the inflammatory bowel diseases autoimmune disorders?", *Gut* **31**, 961–963.

Staag, A.J., Hart, A.L., Knight, S.C. and Kamm, M.A. (2003) "The dendritic cell: its role in intestinal inflammation and relationship with gut bacteria", *Gut* **52**, 1522–1529.

Straub, R.H. and Besedovsky, H.O. (2003) "Integrated evolutionary, immunological, and neuroendocrine framework for the pathogenesis of chronic disabling inflammatory diseases", *FASEB J.* **17**, 2176–2183.

Sugi, K., Saitoh, O., Matsuse, R., Tabata, K., Uchida, K., Kojima, K., *et al.* (1999) "Antineutrophil cytoplasmic antibodies in Japanese patients with inflammatory bowel disease: prevalence and recognition of putative antigens", *Am. J. Gastroenterol.* **94**, 1304–1312.

Sutton, C.L., Kim, J., Yamane, A., Dalwadi, H., Wei, B., Landers, C., *et al.* (2000) "Identification of a novel bacterial sequence associated with Crohn's disease", *Gastroenterology* **119**, 23–31.

Swidsinski, A., Ladhoff, A., Pernthaler, A., Swidsinski, S., Loening-Baucke, V., Ortner, M., *et al.* (2002) "Mucosal flora in inflammatory bowel disease", *Gastroenterology* **122**, 44–54.

Tabaqchali, S., O'Donaghue, D.P. and Bettelheim, K.A. (1978) "*Escherichia coli* antibodies in patients with inflammatory bowel disease", *Gut* **19**, 108–113.

Takahashi, F. and Das, K.M. (1985) "Isolation and characterization of a colonic autoantigen specifically recognized by colon tissue-bound immunoglobulin G from idiopathic ulcerative colitis", *J. Clin. Investig.* **76**, 311–318.

Takeda, K., Kaisho, T. and Akira, S. (2003) "Toll-like receptors", *Annu. Rev. Immunol.* **21**, 335–376.

Taurog, J.D., Richardson, J.A., Croft, J.T., Simmons, W.A., Zhou, M., Fernandez-Sueiro, J.L., *et al.* (1994) "The germfree state prevents development of gut and joint inflammatory disease in HLA-B27 transgenic rats", *J. Exp. Med.* **180**, 2359–2364.

Thayer, W.R. (1976) "Are the inflammatory bowel diseases immune complex diseases?", *Gastroenterology* **70**, 136–137.

Toms, C. and Powrie, F. (2001) "Control of intestinal inflammation by regulatory cells", *Microbes Infect.* **3**, 929–935.

VandeMerwe, J.P., Schroder, A.M., Wensinck, F. and Hazenberg, M.P. (1988) "The obligate anaerobic faecal flora of patients with Crohn's disease and their first-degree relatives", *Scand. J. Gastroenterol.* **23**, 1125–1131.

Vezys, V. and Lefrançois, L. (2002) "Inflammatory signals drive organ-specific autoimmunity to normally cross-tolerizing endogenous antigen", *J. Immunol.* **169**, 6677–6680.

Waldner, H., Collins, M. and Kuchroo, V.K. (2004) "Activation of antigen-presenting cells by microbial products breaks self tolerance and induces autoimmune disease", *J. Clin. Investig.* **113**, 990–7.

Watanabe, T., Kitani, A., Murray, P.J. and Strober, W. (2004) "NOD2 is a negative regulator of toll-like receptor 2-mediated T helper type 1 responses", *Nat. Immunol.*, (advanced on line publication).

Winter, H.S., Crum, P.M., King, N.W., Seghal, P.K. and Roche, J.K. (1989) "Expression of immune sensitization to epithelial cell-associated components in the cotton-top tamarin: a model of chronic ulcerative colitis", *Gastroenterology* **97**, 1057–1082.

Wirtz, S., Finotto, S., Kanzler, S., Lohse, A.W., Blessing, M., Lehr, H.A., *et al.* (1999) "Chronic intestinal inflammation in STAT-4 transgenic mice: characterization of disease and adoptive transfer by TNF- plus IFN-γ-producing CD4+ T cells that respond to bacterial antigens", *J. Immunol.* **162**, 1884–1888.

Wucherpfennig, K.W. (2001) "Mechanisms for the induction of autoimmunity by infectious agents", *J. Clin. Investig.* **108**, 1097–1104.

Young, C.A., Sonnenberg, A. and Burns, E.A. (1994) "Lymphocyte proliferation to baker's yeast in Crohn's disease", *Digestion* **55**, 40–43.

